# Bypassing the Identification: MS2Quant for Concentration Estimations of Chemicals Detected with Nontarget LC-HRMS from MS^2^ Data

**DOI:** 10.1021/acs.analchem.3c01744

**Published:** 2023-08-07

**Authors:** Helen Sepman, Louise Malm, Pilleriin Peets, Matthew MacLeod, Jonathan Martin, Magnus Breitholtz, Anneli Kruve

**Affiliations:** † Department of Materials and Environmental Chemistry, Stockholm University, Svante Arrhenius väg 16, 106 91 Stockholm, Sweden; ‡ Department of Environmental Science, Stockholm University, Svante Arrhenius väg 8, 106 91 Stockholm, Sweden; § Science for Life Laboratory, Department of Environmental Science, Stockholm University, Svante Arrhenius väg 8, 106 91 Stockholm, Sweden

## Abstract

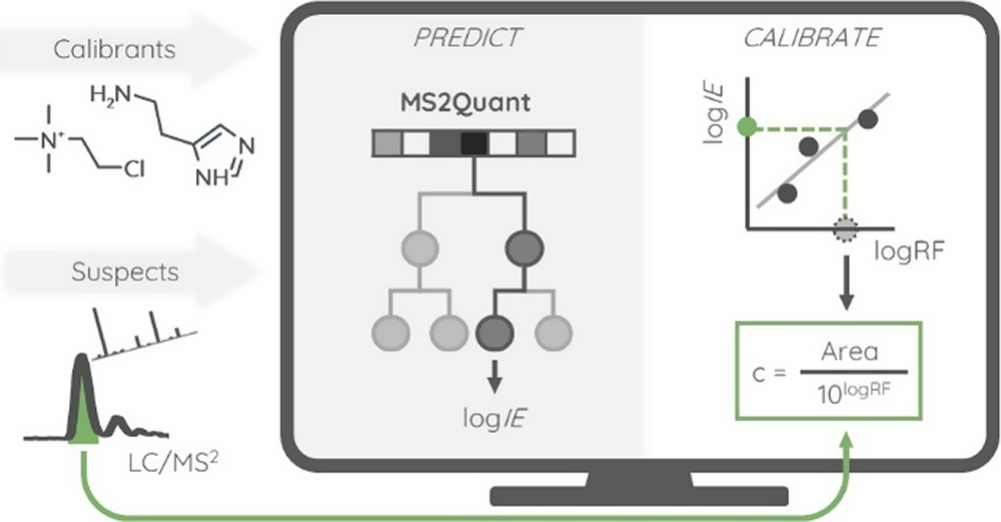

Nontarget analysis by liquid chromatography–high-resolution mass spectrometry (LC-HRMS) is now widely used to detect pollutants in the environment. Shifting away from targeted methods has led to detection of previously unseen chemicals, and assessing the risk posed by these newly detected chemicals is an important challenge. Assessing exposure and toxicity of chemicals detected with nontarget HRMS is highly dependent on the knowledge of the structure of the chemical. However, the majority of features detected in nontarget screening remain unidentified and therefore the risk assessment with conventional tools is hampered. Here, we developed MS2Quant, a machine learning model that enables prediction of concentration from fragmentation (MS^2^) spectra of detected, but unidentified chemicals. MS2Quant is an *xgbTree* algorithm-based regression model developed using ionization efficiency data for 1191 unique chemicals that spans 8 orders of magnitude. The ionization efficiency values are predicted from structural fingerprints that can be computed from the SMILES notation of the identified chemicals or from MS^2^ spectra of unidentified chemicals using SIRIUS+CSI:FingerID software. The root mean square errors of the training and test sets were 0.55 (3.5×) and 0.80 (6.3×) log-units, respectively. In comparison, ionization efficiency prediction approaches that depend on assigning an unequivocal structure typically yield errors from 2× to 6×. The MS2Quant quantification model was validated on a set of 39 environmental pollutants and resulted in a mean prediction error of 7.4×, a geometric mean of 4.5×, and a median of 4.0×. For comparison, a model based on PaDEL descriptors that depends on unequivocal structural assignment was developed using the same dataset. The latter approach yielded a comparable mean prediction error of 9.5×, a geometric mean of 5.6×, and a median of 5.2× on the validation set chemicals when the top structural assignment was used as input. This confirms that MS2Quant enables to extract exposure information for unidentified chemicals which, although detected, have thus far been disregarded due to lack of accurate tools for quantification. The MS2Quant model is available as an R-package in GitHub for improving discovery and monitoring of potentially hazardous environmental pollutants with nontarget screening.

## Introduction

Advances in liquid chromatography (LC) coupled to high-resolution mass spectrometry (HRMS) by electrospray ionization (ESI) have revolutionized the detection of unknown chemicals in the environment. Utilizing advanced computational tools and spectral libraries to identify or tentatively annotate the structure of the detected chemicals has facilitated a shift from targeted analysis toward nontarget screening (NTS). NTS with LC-HRMS enables the detection of previously overlooked and emerging contaminants and their transformation products;^2−6^ however, to assess the risk posed by chemicals in the environment, it is necessary to know both their intrinsic toxicity and concentration.^1,7^

The concentration of an identified chemical can be accurately determined by calibrating to an analytical standard. However, quantification is more challenging if analytical standards are not available as the detected signal intensity is poorly correlated to the concentration across different chemicals. The reason for this is that ionization efficiency in ESI, and therefore the response factor described by the slope of the calibration graph for individual chemicals can differ by orders of magnitude.^8−10^ The electrospray ionization mechanism is ambiguously understood; however, intrinsic properties such as hydrophobicity,^8,11,12^ proton affinity in gas^13^ and liquid phase,^14,15^ as well as properties of the mobile phase such as solvent evaporation rate,^16,17^ pH,^18,19^ and additive type^20^ can influence the response factor of a chemical. In addition, the design of the ESI source^17^ and instrumental parameters^21^ can have an impact on the ionization process.

Determining the response factor of detected chemicals present in the sample is crucial to pinpoint the chemicals posing the highest risk due to high exposure to these substances. Furthermore, these chemicals can be prioritized for identification and quantification. Recently, several approaches for the quantification of chemicals that are tentatively identified with candidate structures in suspect or nontarget screening have been proposed. First, the calibration graph of structurally similar chemicals,^23^ such as parent compound for transformation products^24,25^ or a homologue,^26^ can be used to quantify chemicals detected and identified with NTS. Second, calibration with closely eluting chemicals^27^ may be utilized. Lastly, machine learning models can be trained to predict response factors and this prediction can be further used for quantification.^10,28−35^ Such quantification methods enable the estimation of concentration as well as prioritization and typically have errors from 2× to 6×.^10,25,27−31,36^

Previous machine learning based quantification approaches require that a candidate structure is first assigned from the NTS data processing, then molecular descriptors are computed for this structure using simplified molecular-input line-entry system (SMILES) notation. The molecular descriptors calculated from the structure are model-specific and may include physicochemical properties,^31^ Pharmaceutical Data Exploration Laboratory (PaDEL) descriptors,^10,37^ and/or different structural fingerprints.^36^ Such machine learning models predict relative ionization efficiency for the candidate structures which need to be further translated into instrument- and method-specific response factors with the help of a set of calibration compounds. Most quantification approaches require a single candidate structure that is assumed to be accurate. However, the variation in rates of correctly identified structures is dependent on the workflow, data quality, and available databases.^4,38−41^ For example, Wang et al. detected 335 potential organic micropollutants with suspect and nontarget screening, while 133 candidate structures were successfully confirmed with analytical standards.^38^ Furthermore, the fraction of LC-HRMS peaks (features with retention time and two-dimensional MS information) that remain unidentified generally surpasses the number of annotated peaks.^2,4,42,43^ As an example, Papazian et al.^4^ managed to annotate 17% of 60,300 detected molecular features in air samples from the Indian subcontinent, achieving this high number of annotations by using both liquid and gas chromatography as well as in silico structural predictions. Some of the previously developed quantification approaches allow predictions both for structurally identified as well as unidentified chemicals. For example, Pieke et al.^27^ used close eluting standards to quantify detected chemicals with an error up to 4× while Kruve et al.^24^ observed a mean error of 3.3× and a maximum error of 88×. Additionally, Groff et al.^44^ recently evaluated a bounded response factor method where quantile estimates from the distribution of response factors for standards are used to estimate concentration yielding errors up to 150× for positive mode ESI. Machine learning models taking advantage of empirical analytical information of detected chemicals have the potential to overcome high prediction errors for structure-free quantification. Recently, Palm and Kruve^22^ showed that a combination of retention time, exact mass, and the response ratio of peak areas in positive and negative mode can be used to predict the ionization efficiency of chemicals in surface water samples with a mean error of 10× using a machine learning model. Although promising, the approach requires measuring one sample with three sets of chromatographic conditions, as well as utilizing both positive and negative ESI mode.

MS^2^ spectra carry structurally relevant information about functional groups,^45,46^ which further provide information about the compounds’ polarity as well as acid–base properties. A machine learning model, MS2Tox, was recently developed to predict toxicity (LC_50_ values) of unidentified compounds based on structural fingerprints calculated from the MS^2^ spectrum.^47^ Here, we exploit the same principle and develop a novel quantification approach for unidentified chemicals detected with NTS LC-HRMS using only the mobile phase, MS^1^ and MS^2^ spectra. SIRIUS+CSI:FingerID^41,45,48−50^ was used to predict the probability that structural fingerprints are present in a chemical from measured MS^2^ spectrum. To predict ionization efficiency from the structural fingerprints, a unified dataset of 1191 unique known chemicals compiled from 13 datasets measured on 13 instruments was used. We compare our MS^2^-based quantification approach, MS2Quant, against the best performing comparable candidate structure-based model we could develop, a PaDEL based model, for quantification of 39 chemicals that were used in a NORMAN interlaboratory comparison. We show that ionization efficiency predictions from MS^2^ data are comparable with structure-based predictions and provide a possibility to quantify the exposure of unidentified compounds in LC-HRMS analysis.

## Materials and Methods

### Data for Training the Ionization Efficiency Model

The final dataset contains in total 1191 unique compounds and 6049 datapoints measured with 13 different instruments with different types of electrospray ionization sources representing differences in experimental conditions. The range of log IE values in the final dataset was −1.49 to 7.49. Details about compiling a dataset with unified log IE values can be found in Supporting Information Chapter S1, Code S1, and Tables S1 and S2.

### Calculation of Descriptors

Molecular, structural, and eluent descriptors were used to develop ionization efficiency prediction models. For the development of MS2Quant, a combined set of structural fingerprints (Chemistry Development Kit (CDK) substructure fingerprints, PubChem CACTVS fingerprints, Klekota-Roth fingerprints,^51^ FP3 fingerprints and Molecular ACCess System (MACCS) fingerprints;^52^ altogether 1263 descriptors) was used. This combined set of structural fingerprints (further referred to as “structural fingerprints” for better reading) give information about functional groups in the structure and can be calculated either from structure or from MS^1^ and MS^2^ spectra with SIRIUS+CSI:FingerID identification software. All structural fingerprints for chemicals used in the training and testing of the model were calculated using “*rcdk*” library and define structural keys of different size bits.^53^ Performance of other tested descriptors can be found in Table S3 and Chapter S2. Additionally, eluent descriptors such as organic modifier percentage, aqueous pH, polarity index, surface tension, and viscosity were added to modeling data due to the known strong effect of environment on ionization efficiency in electrospray ionization processes. These descriptors have been shown to have a strong impact on ionization efficiency in prediction models.^10,18−20^

### Data Preprocessing

All features (columns with descriptor values) with more than 10 missing values per descriptor were removed from the dataset, resulting in 633, 1267, 1024, 1024, and 1263 descriptors for PaDEL, Mordred, ECFP2, MAP4, and structural fingerprints, respectively. Similarly, features with near-zero variance were removed from the dataset with the frequency cutoff value of 80/20, leaving 544, 968, 22, 1024, and 184 descriptors for PaDEL, Mordred, ECFP2, MAP4, and structural fingerprints, respectively. Pair-wise correlations were reduced by removing columns with the largest mean absolute correlation in pairs using the correlation cut-off value of 0.75. After preprocessing, the number of descriptors left were 144 for PaDEL, 175 for Mordred, 20 for ECFP2, 630 for MAP4, and 117 for structural fingerprints.

### Modeling Parameters

The ionization efficiency data were divided into the training and test set with a ratio of 80/20, giving a training and test set of 4654 and 1395 datapoints. Splitting was performed based on InChIs to avoid having the same compound measured under different conditions in both sets. Extreme gradient boosting-based algorithms, in which ensemble models are trained additively,^54,55^ were tested as these have found use in modeling with structural fingerprints.^47^ Using *caret* R-package, tree-based (*xgbTree*), linear function (*xgbLinear*), and dropout additive regression trees (*xgbDART*) extreme gradient boosting-based algorithms were tested. The hyperparameters were optimized with the “boot” resampling method using 5-fold cross-validation. Additionally, *y*-randomization analysis was performed to MS2Quant which proved the model predictions to be better than random (RMSE of training and test set of 1.107 (12.8×) and 1.144 (13.9×) log-units, respectively; *R*^2^ of 0.01).

The model performance was evaluated using the root mean square errorof log IE ofthe training and test set as well as mean and median of fold errors that were calculated for each datapoint by the following formula:
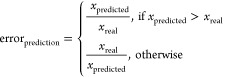
1where *x* corresponds to log IE or concentration for models’ development or validation, respectively. Performance of all developed models can be found in Table S3. Data and codes that were used for modeling can be found on on GitHub (https://github.com/kruvelab/MS2Quant).

### Chemicals Used in Validation and Fingerprint Prediction from MS^2^ Data

Detailed overview of NORMAN interlaboratory comparison, chemicals used in this study, and experimental conditions can be found in Supporting Information Tables S4 and S5 and Chapter S3. Detailed information about how SIRIUS+CSI:FingerID was used for calculating structural fingerprints and identification results can be found in Chapter S4 and Table S8.

### Converting Predicted Response Factor to the Predicted Ionization Efficiency

To convert a predicted ionization efficiency value to an instrument and measurement specific response factor, calibration of the model is performed by measuring calibrants during the same experimental run with suspects. To predict response factor of a suspect chemical, the ionization efficiency is predicted and converted to the response factor using the regression obtained from calibration compounds using the following equation:
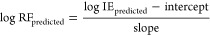
2

## Results and Discussion

### Model Development

**Figure 1:**
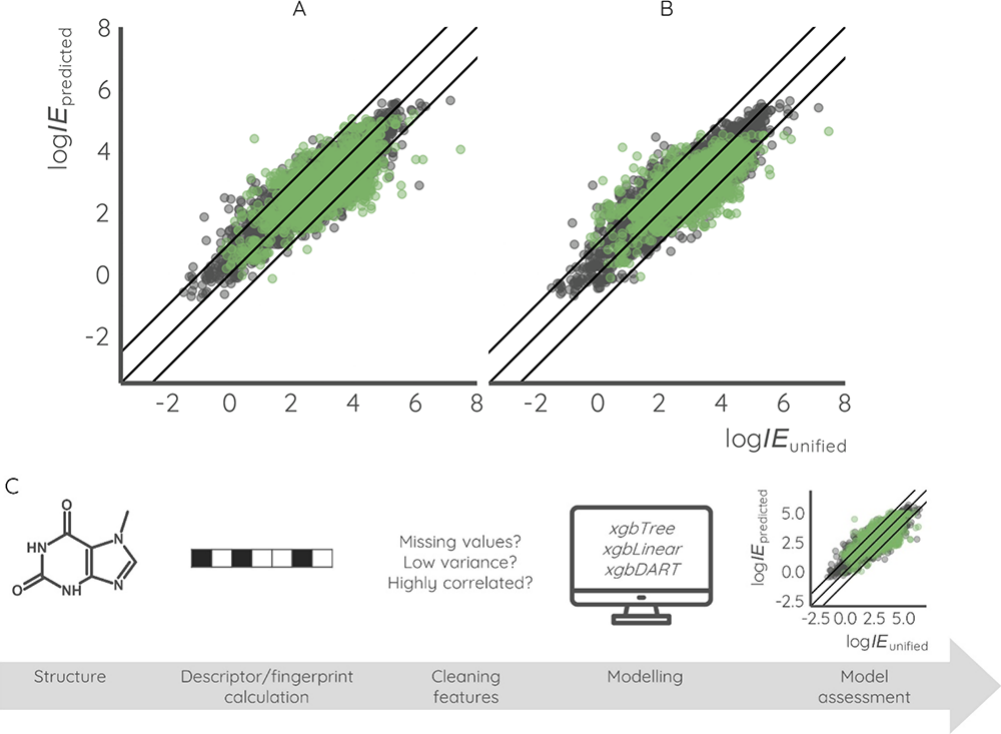
T*raining* (gray) and *test* (green) sets of two best performing models trained with the *xgbTree* algorithm and based on (A) structural fingerprints in MS2Quant and (B) on PaDEL descriptors. (C) General modeling workflow used here. For all 1191 chemicals, molecular descriptors/fingerprints were calculated from the structure and 80% of the data (training set) was used for modeling. To clean the descriptors, features with more than 10 missing values were removed. Additionally, features with near-zero variance (cut-off 80/20) and pair-wise correlation (cut-off 0.75) were removed. The training set chemicals were then used for modeling and the performance was assessed based on RMSE and fold prediction errors of the test set.

MS2Quant has the advantage of estimating concentrations for both identified and unidentified chemicals from nontarget LC-HRMS analysis. In the case of a known or tentatively identified structure, the SMILES notation of a chemical can be used to calculate the structural fingerprints. For unidentified chemicals, the MS^2^ spectra are first used to predict the probability of presence or absence of structural fingerprints, thereby providing insight into properties of the chemical.^41,47^ To evaluate the suitability of structural fingerprints for predicting the ionization efficiency, we trained and validated the MS2Quant model based on structural fingerprints and eluent descriptors. For model training, the SMILES notation of 952 chemicals was used to calculate associated structural fingerprints, and three machine learning algorithms were used for training (*xgbTree*, *xgbLinear,* and *xgbDART*). The models’ performances were evaluated based on a test set of 239 previously measured chemicals. The highest predictive power on the test set was observed for the *xgbTree* training algorithm. MS2Quant resulted in root mean square errors (RMSE) of 0.55 and 0.80 log-units for the training and test set, respectively. These RMSE values correspond to 3.5× and 6.3× fold errors; see Figure 1A. The mean, geometric mean, and median prediction errors for the test set calculated based on eq 1 were 15.4×, 4.3×, and 3.2×, respectively. This provides significant advances in ionization efficiency predictions compared to the whole range of 100,000,000× for ionization efficiency values within the training dataset used in this work.

In comparison, a previously published prediction model by Liigand et al.^10^ resulted in root mean square errors of 1.9× and 3.0× on the training and test set, respectively. The latter model included 3139 datapoints measured under various eluent compositions and was validated on a set of 35 chemicals with a mean prediction error of 5.4×; however, a direct comparison with the model is impossible as MS2Quant is trained on a significantly larger, more heterogenous, set of chemicals (*n* = 954 vs *n* = 353) from 13 datasets. To evaluate the impact of selection of molecular features on ionization efficiency prediction accuracy, different models using molecular features were trained. Namely, models using PaDEL, Mordred, ECFP2, and MAP4 descriptors were considered. The highest predictive power was observed for PaDEL descriptors with the *xgbTree* training algorithm, with a RMSE for the training set of 0.56 log-units (3.6×) and the RMSE for the test set of 0.81 log-units (6.5×); see Figure 1B. The mean, geometric mean, and median prediction errors calculated for the test set by eq 1 were 11.7×, 4.4×, and 3.6×, respectively. The difference in the performances of the latter and MS2Quant models was insignificant. This indicates that structural fingerprints can provide similar information about the ionization efficiency of the chemicals as the continuous or hashed molecular features. For a comprehensive comparison of all trained models, please see Table S3.

In principle, both PaDEL descriptors and structural fingerprints (MS2Quant) have a similarly good starting point for ionization efficiency predictions due to the overlap in information incorporated by both features. For example, PaDEL descriptors include information about numbers of hydrogen bond donors and acceptors, solute hydrogen bond basicity and acidity, and atom counts, e.g., for nitrogen which is often the favored protonation site. Certain functional groups described by structural fingerprints in MS2Quant may account for similar information, such as carbonyl or primary, secondary, and tertiary amines, and therefore structural information is similarly beneficial for predicting the ionizability of a compound.

For validation of MS^2^ spectra-based quantification, the test and training sets were merged and an updated MS2Quant model was trained. Also, a new model based on PaDEL descriptors for structure-based quantification was trained. The model trained on all datapoints using the respective previously optimized hyperparameters is available in the *MS2Quant* R-package in GitHub.

### MS2Quant Performance in NTS Workflow on NORMAN Interlaboratory Comparison Samples

MS2Quant was validated under environmentally relevant conditions. Briefly, the surface water matrix was spiked with a mixture of relevant water pollutants covering ionization efficiency values over more than four orders of magnitude with the peaks spread out over the whole reverse phase chromatography run. MS^2^ data were acquired in data-dependent acquisition mode with a target inclusion list. The calibration solutions and high and low concentration spiked lake water samples were obtained from NORMAN interlaboratory comparison on quantification in NTS LC-HRMS.^56,57^

**Figure 2:**
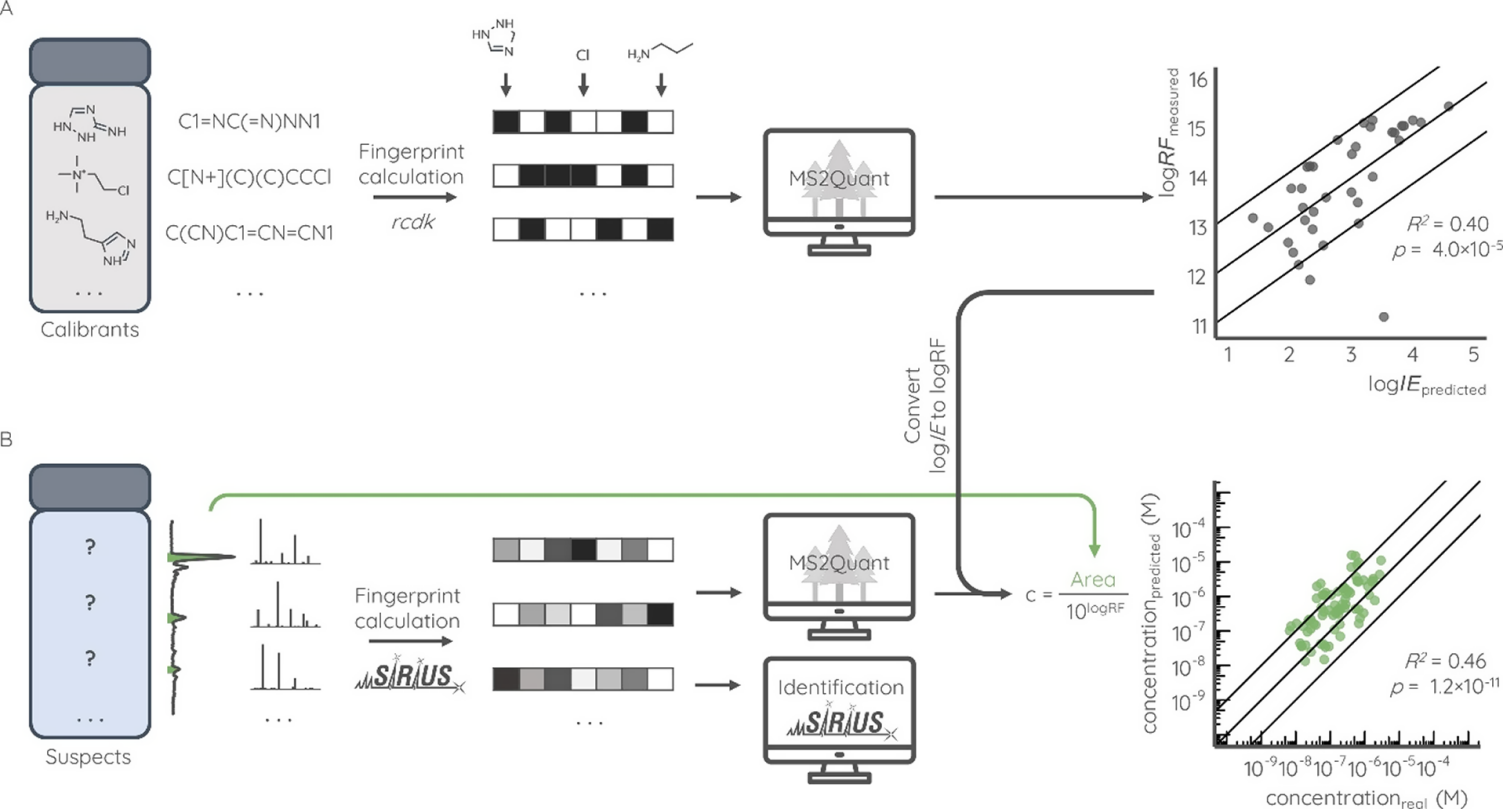
Workflow for validation of MS2Quant on NORMAN interlaboratory comparison samples. (A) Molecular fingerprints were computed for 36 chemicals in the calibration mix from SMILES notation with the *rcdk* package in R. Furthermore, MS2Quant was used to predict ionization efficiency values and linear regression was fit between experimental logarithmic response factors and logarithmic predicted ionization efficiencies. (B) Lake water spiked with 39 suspect compounds in high and low concentrations was measured with LC-HRMS in data-dependent acquisition mode with an inclusion list. SIRIUS+CSI:FingerID was used to predict probabilities of structural fingerprints from MS^1^ and MS^2^ spectra and MS2Quant was used to predict ionization efficiencies from these predicted probabilities. Thereafter, the linear regression from calibration compound was used to convert the predicted ionization efficiency values to instrument- and method-specific predicted response factors. Concentrations of suspect chemicals were found using predicted response factors as well as integrated areas from LC-HRMS analysis and was compared to the spiked concentrations. For comparison with PaDEL-based quantification, a similar workflow was used with the PaDEL descriptor-based prediction model instead of MS2Quant and identification of suspects was performed with SIRIUS+CSI:FingerID where the top assigned structure was used for ionization efficiency predictions.

For the 36 calibration compounds, molecular fingerprints were computed from SMILES and used to predict ionization efficiency with MS2Quant. Only chemicals observed as protonated molecules or permanently positively charged were considered, as all the training data for predictive ionization efficiency model use these ions exclusively. Measured response factors and predicted ionization efficiency values were correlated (*R*^2^ = 0.40, *p* = 4.0 × 10^–5^) with a residual standard error of 0.85; see Figure 2A.

The MS^2^ spectra were recorded for 39 suspects and were used alongside MS^1^ spectra to predict the probability of structural fingerprints with SIRIUS+CSI:FingerID^41^ for each chemical. Thereafter, the fingerprints were used to predict the ionization efficiency of the chemicals which were converted to predicted response factor values. The suspects were quantified in two lake water samples from NORMAN interlaboratory comparison, spiked at high and low concentrations. The predicted concentrations ranged from 1.3 × 10^–8^ to 1.6 × 10^–5^ M and were similar to the real spiked concentrations for the suspects with the range of 6.6 × 10^–9^–2.9 × 10^–6^ M.

For validation, the predicted concentrations were compared to spiked concentrations. Generally, the estimated concentrations were over-predicted, as for 78% of datapoints, the predictions exceeded the real concentrations; see Figure 2B. The RMSE between real and predicted concentrations was 5.9×, which was lower than the RMSE of the test set observed for MS2Quant model development (6.3×). The mean prediction error observed for MS2Quant was 7.4×, geometric mean 4.5×, and median error 4.0×, indicating similar performance to the model developed by Liigand et al.^10^ which had 5.4× mean prediction error for a validation set of 35 compounds. The compounds with the highest prediction error were omethoate (47.7× and 38.2× for low and high spike, respectively) and metformin (44.6× and 27.8× for low and high spike, respectively).

It is important to note that 26 validation set chemicals were also present in the final MS2Quant training data. For the 13 validation set chemicals that were not present in the training set of MS2Quant, the mean, geometric mean, and median errors were 5.5×, 4.0×, and 3.9×, respectively. Meanwhile, for the 26 chemicals present in the model, the respective errors were 8.3×, 4.7×, and 4.0×. The errors are slightly smaller for chemicals that were not present in the model; however, the differences are minor.

#### Comparison of MS^2^-Based Quantification and Suggested Structure-Based Quantification

MS2Quant was compared with the PaDEL-based ionization efficiency prediction model developed here, which uses the structural assignment as the basis for quantification. The PaDEL ionization efficiency prediction model was trained on the same chemical space as MS2Quant to allow a fair comparison. Additionally, the structure-based model developed by Liigand et al.^10^ was used to compare the application domains. For this, the structural assignments were generated for each suspect LC-HRMS peak with SIRIUS + CSI:FingerID using the same MS^2^ spectra that were previously used as input for MS2Quant. Based on the SMILES of the top structural candidate, PaDEL descriptors were computed and used to predict the ionization efficiencies of the structural candidates and the ionization efficiencies were further used to predict the concentrations assuming that the ionization efficiency of the top structure represents the correct chemical. The summary models’ performances on the validation set and a graphical comparison can be found in Tables S6 and S7.

In general, a slightly higher mean, geometric mean, and median error of 9.5×, 5.6×, and 5.2×, respectively, were observed for PaDEL-based quantification when using the top assigned structure as input; however, based on the pair-wise Wilcoxon ranked sum exact test, the difference in quantification errors were statistically insignificant (*p*-value = 0.13). A similar performance was observed for MS2Quant as well as the model developed by Liigand et al. when top assigned structures were used as input; see Table 1. It is important to note that the training data used to develop MS2Quant- and PaDEL-based model included 27 validation set compounds, while the training data for the model developed by Liigand et al.^10^ only included 4 validation set chemicals, which can explain some differences in the performances of these models.

**Table 1:** Comparison of the Performance of Four Quantification Models on the Validation Set Suspect Compounds Spiked in the Surface Water^a^

		MS2Quant (MS^2^)	MS2Quant (structure)	PaDEL-based model developed here (954 chemicals)	PaDEL-based model developed by Liigand et al. (353 chemicals)
results of “true NTS” (MS2Quant from MS^2^, others with top suggested structure) (39 chemicals)	RMSE	5.85	7.29	7.42	7.26
	*R*^2^	0.46	0.37	0.47	0.49
	Mean	7.40	9.51	9.51	8.99
	Geom. mean	4.45	5.44	5.63	5.40
	Q25	2.16	2.48	2.29	2.27
	Q50 (Median)	4.02	4.57	5.19	4.87
	Q75	8.27	10.54	12.74	13.08
	Q90	17.43	25.31	26.09	20.36
	Q100 (Max)	47.68	55.26	54.91	45.87
correct SMILES is used for quantifying suspects (39 chemicals)	RMSE		6.77	7.05	7.99
	*R^2^*		0.42	0.54	0.46
	Mean		8.18	8.61	9.89
	Geom. mean		5.29	5.44	6.00
	Q25		2.42	2.60	2.35
	Q50 (Median)		5.78	5.49	6.56
	Q75		9.71	9.47	15.20
	Q90		20.78	23.69	20.87
	Q100 (Max)		38.73	35.87	55.26
only suspects that were correctly identified (34 chemicals)	RMSE	6.12	7.57	7.64	7.34
	*R*^2^	0.43	0.34	0.44	0.48
	Mean	7.80	9.91	9.81	8.83
	Geom. mean	4.67	5.69	5.83	5.52
	Q25	2.28	2.53	2.37	2.30
	Q50 (Median)	4.09	5.27	5.20	6.61
	Q75	8.36	10.67	13.35	13.09
	Q90	17.95	25.78	26.09	19.48
	Q100 (Max)	47.68	55.26	54.91	41.25
only suspects that were incorrectly identified (5 chemicals)	RMSE	4.15	5.51	6.02	6.73
	*R*^2^	0.68	0.61	0.65	0.55
	Mean	4.66	6.78	7.46	10.09
	Geom. mean	3.20	4.00	4.47	4.63
	Q25	1.72	1.99	1.71	2.29
	Q50 (Median)	2.45	2.96	5.12	3.45
	Q75	4.70	6.68	6.41	4.91
	Q90	11.02	17.64	19.24	32.19
	Q100 (Max)	15.71	25.14	27.42	45.87
only incorrectly identified suspects, but the correct SMILES was used for quantification (5 chemicals)	RMSE		4.39	6.29	6.77
	*R*^2^		0.59	0.72	0.54
	Mean		4.93	7.06	8.52
	Geom. mean		3.38	4.90	5.41
	Q25		1.84	2.00	2.67
	Q50 (Median)		2.16	6.94	4.69
	Q75		6.14	9.46	7.64
	Q90		11.04	12.97	21.90
	Q100 (Max)		15.73	18.48	31.21

a The quantification was performed with MS2Quant using MS^1^ and MS^2^ spectra as input, MS2Quant using SMILES notation as input, PaDEL-based model developed in this work using SMILES as input and PaDEL based model developed by Liigand et al.^10^ using SMILES notation as the input.

Out of 39 suspect compounds for which fragmentation spectra were acquired in DDA, 34 compounds were identified correctly as top assigned structures. Two detected LC-HRMS peaks belonging to carbamazepine-10,11-epoxide and 5-chlorobenzotriazole had the correct structure ranked second. For two other peaks corresponding to sebuthylazine and dazomet, the correct structure ranked third. Additionally, one peak corresponding to sudan I was correctly identified only as top 223 structure. The correct and assigned top structures can be found in Table S8.

For five chemicals with incorrectly assigned top structure, the mean, geometric mean, and median prediction error with MS2Quant calculated from MS^2^ were lower compared to other models; however, the differences were statistically insignificant according to the pair-wise Wilcoxon rank sum exact test (*p*-value = 0.44). MS^2^ spectra-based quantification results with MS2Quant ranged between 1.2× and 15.7×, with the PaDEL-based model developed here between 1.3× and 27.4× and with model developed by Liigand et al. between 1.4× and 45.9×.

**Figure 3:**
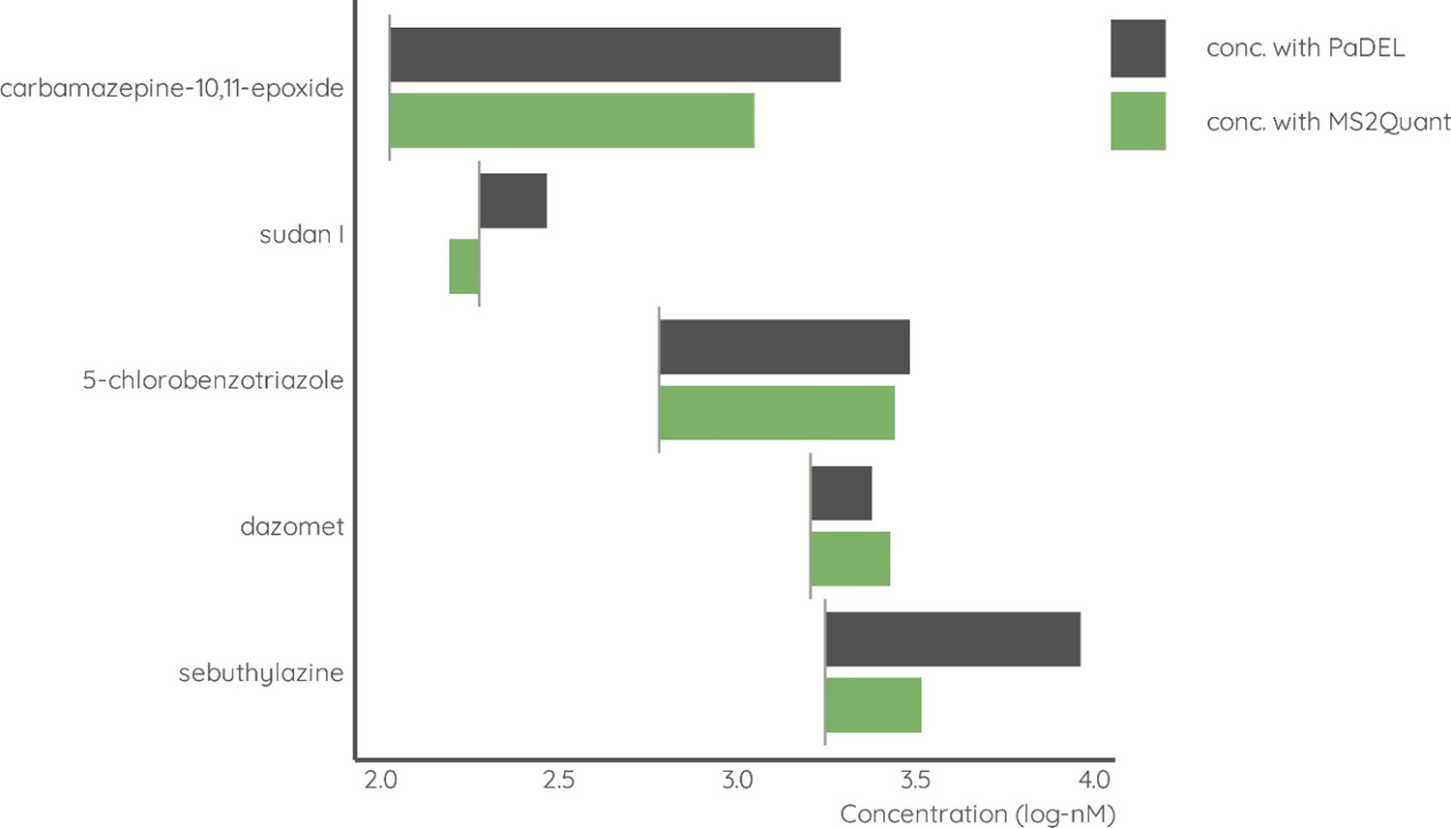
Predicted concentrations for high concentration spiked sample with MS2Quant and the PaDEL-based model for five incorrectly identified compounds. Real concentrations are marked with a vertical line.

Generally, in five cases of incorrect structural assignment, both MS2Quant and the PaDEL-based model developed here over-estimated the concentrations, see Figure 3. Still, MS2Quant yielded concentrations closer to spiked concentrations in four out of five cases. Only for dazomet a lower prediction error was observed with the PaDEL-based prediction model even when using the wrong structural assignment. Using PaDEL descriptors of the wrong structural assignment of dazomet yielded a 1.8× error while MS2Quant yielded an error of 2.1×. The results from validation indicate that incorrect structural assignment can yield similar or higher prediction error compared to using MS^2^ spectra directly for quantification. In general, the performance of MS2Quant that is independent of results of identification is comparable to structure-based methods in use.

### Analysis of Key Features Learned by MS2Quant

**Figure 4:**
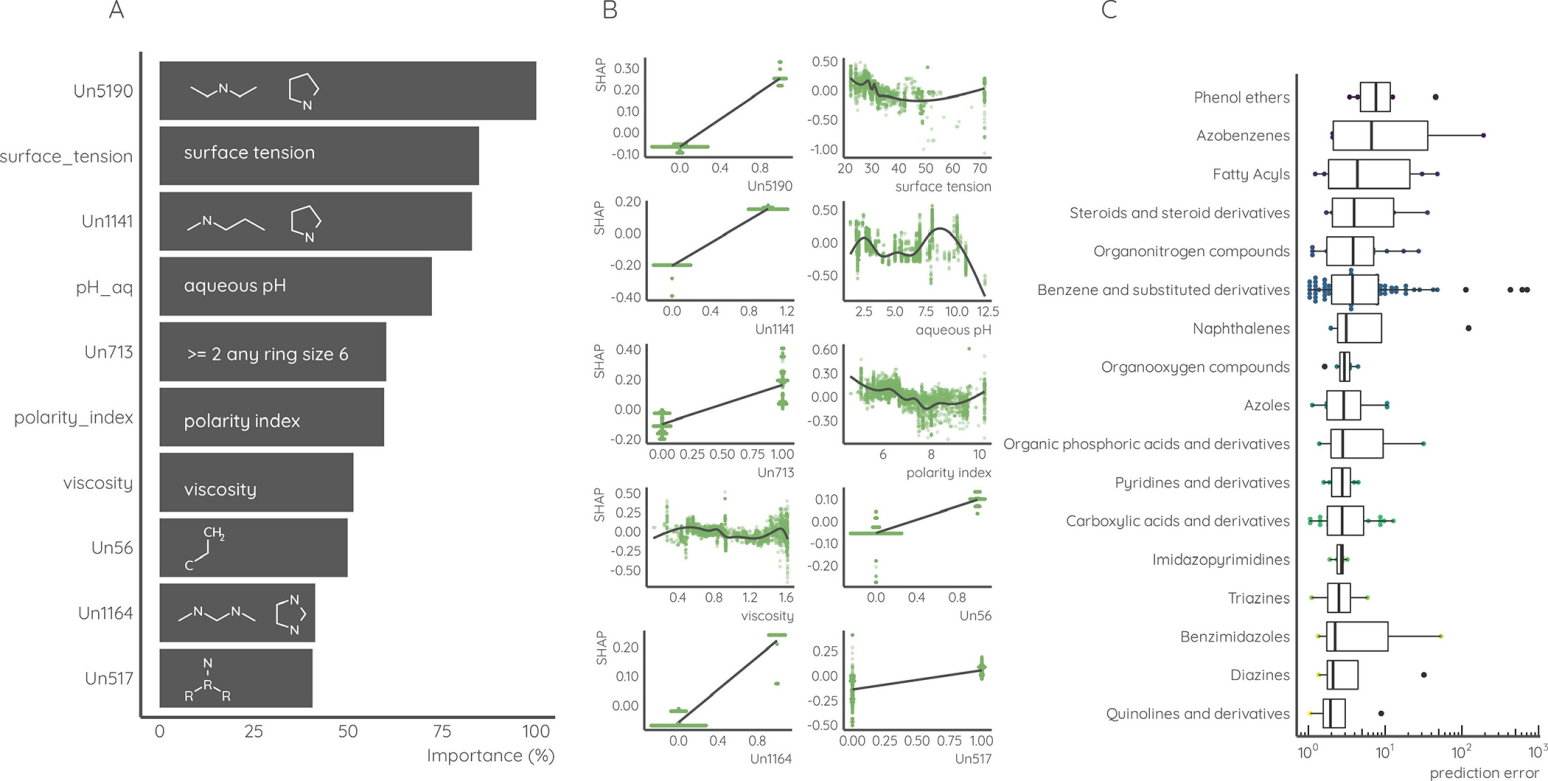
(A) Top 10 most influential variables in the model and their normalized importance (%); (B) SHAP values representing influence of each top 10 feature and their marginal contribution to the prediction and (C) the test set chemicals assigned to different classes by ClassyFire, where each datapoint represents the geometric mean prediction error of log IE of a unique chemical. The classes are in the descending order based on median geometric mean prediction error of all compounds in the group and only classes with three or more unique representatives were plotted.

The features with highest importance for MS2Quant ionization efficiency predictions were investigated using variable importance and SHapely Additive exPlanations (SHAP) values. Firstly, the eluent descriptors are significant as all four descriptors (polarity index, surface tension, viscosity, and pH of the aqueous phase) were among the top 10 most impactful features. As seen in Figure 4A,B, the lower polarity index and surface tension result in higher ionization efficiency, which agrees with previously reported findings by Liigand et al.^10^ for a PaDEL descriptor-based model. Although continuous eluent descriptors offer more potential splits for tree-based algorithms compared to binary fingerprints which can result in higher variable importance, eluent descriptors were also seen as the top 10 most impactful features for the PaDEL-based model trained here; see Table S9 and Figure S1 for detailed analysis. This similarity in feature importance facilitates that accounting for mobile phase composition in ionization efficiency predictions is of high importance, as also observed previously.^18,19,29^ Second, chemical properties associated with basicity of the chemical were among the 10 most impactful features. This is visible through nitrogen containing fingerprints referring to basic functional groups that, when present, facilitate a higher predicted ionization efficiency value. This is also known from previous studies where chemicals which are already charged in the mobile phase tend to have higher ionization efficiency.^58^ Third, two fingerprints describing more than two six-member rings and secondary carbon were influential in predicting the ionization efficiency. These fingerprints are possibly accounting for hydrophobicity of the compound. Generally, previous studies show that chemicals with larger hydrophobic moieties possess higher ionization efficiency both in positive^10^ and negative mode.^35^ It is important to mention that in the case of the presence of a structural fingerprint that describes more than one functional group, any of the functional groups are possible and the exact structure cannot be deducted.

To understand the impact of chemical properties to the prediction accuracy, ClassyFire^59^ was used for automated classification of chemicals in the test set. This resulted in classification of test set compounds into 14 superclasses and 121 classes. The geometric mean prediction error was calculated for each unique test set chemical and their distribution for all classes with three or more unique representatives can be seen in Figure 4C. Analysis of 17 classes with three or more representative chemicals showed significant differences between classes based on the Kruskal–Wallis rank sum test (*p*-value = 2.2 × 10^–16^). A pairwise comparisons using the Wilcoxon rank sum test with continuity correction indicated significant differences between all groups except for azobenzenes and fatty acyls. The median prediction error was lower than 10× for all 17 classes.

#### Limitations

The MS2Quant quantification tool was developed to enable concentration estimations for unidentified chemicals based on information that can be extracted from MS^2^ data. This approach uses SIRIUS+CSI:FingerID to estimate probabilities of presence or absence of structural fingerprints. In order to extract meaningful structural information, the MS^2^ spectra that are used as input to SIRIUS must include high mass accuracy data and contain sufficient number of peaks which can be achieved by averaging fragmentation spectra over multiple collision energies.^41^

Depending on the sample, matrix effects can occur and affect the response of the chemicals. In target analysis, this could be corrected by isotope labeled standards that match the analyte; however, this is impossible for unknown chemicals. In a previous study by Wang et al.^60^ it was observed that the model prediction error significantly dominates over the error arising from the matrix effects.

In order to use MS2Quant for quantification, a set of calibration chemicals that cover a wide range of ionization efficiencies needs to be measured together with the sample. MS2Quant can be used to estimate the concentration within the chemical space and ionization efficiency range of training set chemicals used in modeling.

## Conclusions

A concentration prediction model MS2Quant was developed to estimate the exposure to unidentified chemicals detected with LC-HRMS. MS2Quant was tested and validated on positive mode electrospray ionization data from NORMAN network’s interlaboratory comparison and an accuracy comparable to structure-based methods was observed. The future prospects include development and validation of a complementary negative mode electrospray ionization efficiency prediction model to allow exposure estimations in both ESI modes.

Implementation of MS2Quant in NTS workflow allows giving an estimation for exposure of unidentified chemicals that are otherwise discarded from the analysis. Furthermore, it can be used for pinpointing new, emerging contaminants in risk-based prioritization in a rapid manner alone or in combination with toxicity evaluation. Recently, a MS2Tox machine learning model has been proposed by our group to aid fish LC_50_ predictions. In combination, exposure and toxicity predictions can be used to evaluate the risk of each chemical detected with LC-HRMS. In the future, it is of interest to evaluate if the LC-HRMS peaks with the highest risk are identified in NTS and use the predicted risk to gear the identification toward peaks with the highest impact on the total risk possessed by the sample. To use MS2Quant for quantification, a set of calibrants needs to be measured together with the sample to relate the predicted ionization efficiencies to instrument- and method-specific response factors. By providing experimental data for calibrants and unidentified chemicals, LC-HRMS features can be quantified using the pretraied MS2Quant model. This novel quantification method is openly available as an R-package *MS2Quant* on GitHub (https://github.com/kruvelab/MS2Quant).

## Supporting Information Available

Data unification process in detail; example code how the data were unified based on either dataset 1 or a unified dataset; overview of datasets containing metadata and ionization efficiency information used for modeling; comparison between all tested molecular descriptors or fingerprints and machine learning algorithms; overview of the calibrants and suspects used in validation and experimental conditions; a statistical and graphical overview of MS2Quant and structure-based models’ performances on the validation set; overview of incorrectly identified structures and their highest ranked assigned structure by SIRIUS+CSI:FingerID; SIRIUS calculations and parameters used; top 10 most influential variables in a PaDEL-based model developed here; top 10 most influential variables, their SHAP values, and error distribution of different chemical classes assigned by ClassyFire for PaDEL-based model developed here; and first decision three of xgbTree algorithm-based models developed using structural fingerprints and PaDEL descriptors (PDF)
