# Supplementary material for: Bypassing the Identification: MS2Quant for Concentration Estimations of Chemicals Detected with Nontarget LC-HRMS from MS2 Data
Source: Anal Chem. 2023 Aug 7;95(33):12329–38. doi: 10.1021/acs.analchem.3c01744 (PMC10448440; doi:10.1021/acs.analchem.3c01744)
Supplement: Supplementary file 1 — ac3c01744_si_001.pdf [file ac3c01744_si_001.pdf]

Supporting information

Bypassing the identification: MS2Quant for concentration estimations of chemicals detected with nontarget LC-HRMS from MS^2 data

Helen Sepman, [a,b] Louise Malm, [a] Pilleriin Peets, [a] Matthew MacLeod, [b] Jonathan Martin,[c] Magnus Breitholtz [b] and Anneli Kruve [a,b]*

[a] Department of Materials and Environmental Chemistry, Stockholm University, Svante Arrhenius väg 16, 106 91 Stockholm, Sweden

[b] Department of Environmental Science, Stockholm University, Svante Arrhenius väg 8, 106 91 Stockholm, Sweden

[c] Science for Life Laboratory, Department of Environmental Science, Stockholm University, Svante Arrhenius väg 8, 106 91 Stockholm, Sweden

Table of Content

Chapter S1. Data for training the ionization efficiency model S2
Figure S1. Workflow for data unification based on dataset1 ..... S3
Code S1. Example code for data unification based on dataset1 and a unified dataset. S4
Table S1. Unified ionization efficiencies using dataset1 ..... S5
Table S2. Unified ionization efficiencies using the unified dataset. S6
Table S3. All trained models' performance summary. S7
Chapter S2. Summary of all tested descriptors S8
Chapter S3. NORMAN interlaboratory comparison and compounds S9
Table S4. Calibration compounds and isotope labelled standards S10
Table S5. Suspect compounds S11
Table S6. Summary table of the performance of all tested models on validation set. S12
Table S7. Calibration and summary plots of the performance of all tested models on validation set. S13
Table S8. Falsely identified compounds and their top suggested structure S14
Chapter S4. SIRIUS+CSI:FingerID for calculating structural fingerprints and identification S15
Table S9. Top 10 most influential variables learned by PaDEL based model developed here. S16
Figure S2. PaDEL-based model analysis. S17
Figure S3. First tree of xgbTree models S17
References S18

Chapter S1. Data for training the ionization efficiency model

Response factors in ESI positive mode were explored due to ample availability of training data. Thirteen datasets (dataset1 – dataset13) containing experimental response factors were collected from previous studies.[1–15] All datasets consist of calibration graphs where the slope on the graph in the linear range was used as a measure of response factor. To combine all response factors into one dataset, the response factors were transferred to a unified ionization efficiency scale using dataset1 as the basis.[15] Dataset1 has 353 compounds anchored to a relative ionization efficiency scale with methyl benzoate as the zero reference point. It is important to note that all ionization efficiency values in this work are given on a relative scale and will further be referred to as "ionization efficiencies" for better reading.

In short, the common compounds (based on InChI) between dataset1 and a dataset subject to transferring, were used to establish a linear regression between the response factors of the two datasets. Only compounds measured in MeCN/MeOH under similar eluent conditions were used. In this work, up to 30% difference in organic modifier percentage and one-unit difference in pH were considered as sufficiently similar eluent conditions as under these conditions, the expected differences in ionization efficiency are <0.5 log-units.[4] The exception was dataset5, where all compounds were measured at pH = 7.0 and the pH requirement was not set. All response factor values were log-transformed, then a generalized additive model (from *mgcv* package in *R*) was used for linear model fitting and transferring the rest of the measured response factors into ionization efficiency values. The unified ionization efficiency values were determined through data interpolation as the linearity assumption may not hold outside of the tested area; therefore, datapoints that were out of the linear range were discarded (Figure S1).

As the size and represented chemical groups of datasets varied, five datasets had less than ten compounds in common with dataset1. Therefore, the unification was done using all previously unified datasets together as the new unification basis, increasing the number of common compounds measured under similar conditions. The exception was dataset7, which consisted of derivatized amino acids and for which using the unified dataset did not increase the number of common chemicals; therefore, the three chemicals that were common with dataset1 were used for unification. The same workflow shown on Figure S1 was used; however, unified dataset was used as basis for transferring response factors to ionization efficiency values instead of dataset1.

For establishing the raltionship between the response factors (log*RF*) from any dataset and the ionization efficiency (log*IE*) values in dataset1, simplistically, a generalized linear model was fitted described by the following equation:

$$logRF = a * logIE + b \qquad \textit{Eq. S1.}$$

where a and b are slope and intercept, respectively, from generalized linear model fitting.

For the chemicals present only in the dataset subject to transferring, log*RF* was converted to $\log IE_{\mathrm{transferred}}$ by applying the previously fitted linear equation resulting in the following equation:

$$logIE_{transferred} = \frac{logRF - b}{a} \qquad \text{Eq. S2.}$$

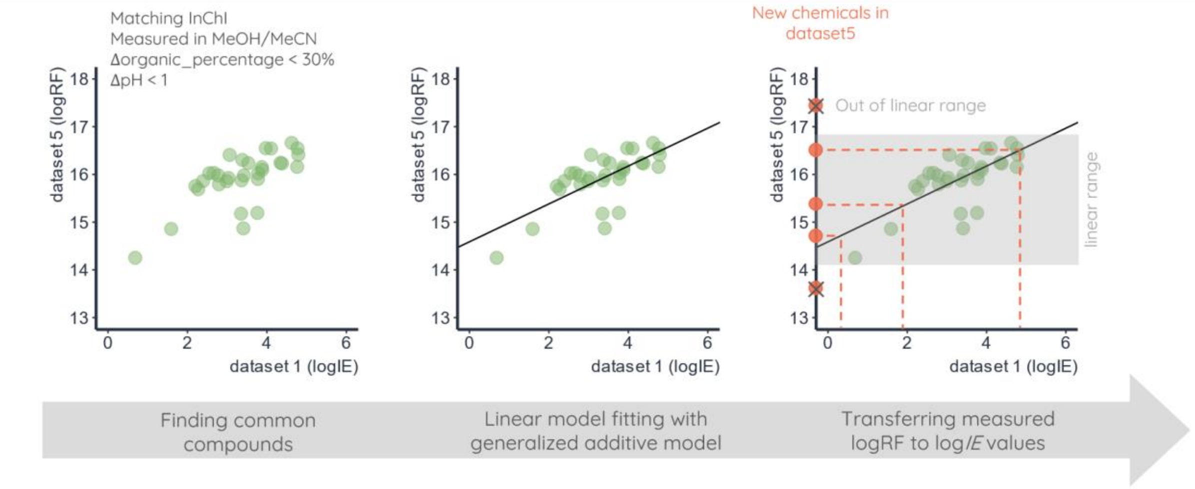

Figure S1. Workflow for data unification based on dataset1. First, common chemicals were found based on matching InChI and similar experimental conditions. Then, linear model was fitted and used to transfer new chemicals from dataset subject to unification into logIE scale. Chemicals out of linear range were discarded. The same workflow was repeated when unified dataset was used as basis instead of dataset1.

Code S1. Example code for data unification based on dataset1 and a unified dataset.

```
#---- transferring dataset2 to dataset1 scale ----
common_dataset1_dataset2 <- all_datapoints_pairs %>%
  filter(delta_per_cent_organic_modifier < 30 & delta_pH < 1) %>%
  filter(Lab == "dataset2",
         Lab1 == "dataset1") %>%
  group_by(inchi, Lab, Lab1) %>%
  summarize(logRF = mean(logRF),
            logRF1 = mean(logRF1)) %>%
  ungroup() %>%
  mutate(Common = TRUE)

regressor_dataset2 <- gam(logRF ~ logRF1,
                          data = common_dataset1_dataset2)

data_dataset2 <- all_data %>%
  filter(Lab == "dataset2") %>%
  filter(logRF < max(common_dataset1_dataset2$logRF) &
         logRF > min(common_dataset1_dataset2$logRF))

data_dataset2 <- data_dataset2 %>%
  mutate(unified_IEs = (logRF - regressor_dataset2$coefficients[1])/
                        regressor_dataset2$coefficients[2])

data_dataset2 <- data_dataset2 %>%
  mutate(unified_IEs = as.numeric(unified_IEs)) %>%
  filter(unified_IEs < max(common_dataset1_dataset2$logRF1) &
         unified_IEs > min(common_dataset1_dataset2$logRF1)) %>%
  unique()

#---- Adding all so far unified IE data together ----

data_unified <- data_dataset1 %>%
  bind_rows(data_dataset2) %>%
  drop_na(unified_IEs)

data_unified <- data_unified %>%
  mutate(logRF = unified_IEs,
         Lab = "Unified")

#generating new all_datapoints_pairs using unified dataset as one of the labs


#---- transferring dataset3 to so far unified dataset scale ----

common_unified_dataset3 <- all_datapoints_pairs %>%
  filter(delta_per_cent_organic_modifier < 30 & delta_pH < 11) %>%
  filter(Lab == "dataset3",
         Lab1 == "Unified") %>%
  group_by(inchi, Lab, Lab1) %>%
  summarize(logRF = mean(logRF),
            logRF1 = mean(logRF1))%>%
  ungroup() %>%
  mutate(Common = TRUE)

regressor_dataset3 <- gam(logRF ~ logRF1,
                          data = common_unified_dataset3)

data_dataset3 <- all_data %>%
  filter(Lab == "dataset3") %>%
  filter(logRF < max(common_unified_dataset3$logRF) &
         logRF > min(common_unified_dataset3$logRF))

data_dataset3 <- data_dataset3 %>%
  mutate(unified_IEs = (logRF- regressor_dataset3$coefficients[1])/
                        regressor_dataset3 $coefficients[2])

data_dataset3 <- data_dataset3 %>%
  mutate(unified_IEs = as.numeric(unified_IEs)) %>%
  filter(unified_IEs < max(common_unified_dataset3$logRF1) &
         unified_IEs > min(common_unified_dataset3$logRF1)) %>%
  unique()
```

Table S1. Unified ionization efficiencies using dataset1. The table includes information about number of unique chemicals by InChI (#chemicals), number of measured datapoints under different conditions (#datapoints), number of chemicals common with dataset1 by InChI and their logIE range (#common with dataset1), lowest and highest unified ionization efficiency value obtained after unification (Lowest logIE, Highest logIE), the variance between the common datapoints (σ^2), comments and references.

Dataset	#chemicals	#datapoints	#common with dataset1	Lowest logIE	Highest logIE	Variance (σ^2)	Comment on compounds	Ref.
dataset1	399	2970	399	-1.49	7.49	0	In-house measured dataset.	[1–5,16]
dataset2	174	580	56 (0.23 – 5.79)	0.31	5.39	0.22		[12]
dataset3	152	193	38 (1.51 – 5.54)	1.52	5.18	0.12		[12]
dataset4	233	233	34 (1.43 – 5.12)	1.45	5.11	0.10		[6]
dataset5	473	478	31 (0.69 – 4.79)	0.69	4.78	0.16	All compounds were measured under pH = 7; therefore, pH was not used for matching. Five compounds have same SMILES and InChI but name (as salt) and other data slightly different.	[17]
dataset6	56	56	13 (1.33 – 4.39)	1.50	4.35	1.05	Only "formic acid" as additive, filtered out pH = 2.91. Removed ibuprofen	[7]
dataset7	20	20	3 (2.86 – 3.95)	2.60	3.60	0.02	Derivatized amino acids; therefore, only 2 common compounds but the dataset was previously anchored with tetraethyl-ammonium.	[10]
dataset8	66	612	15 (1.69 – 4.64)	1.69	4.63	0.17	Only compounds measured with "formic acid" as additive were considered as common for unification.	[13]
dataset9	67	581	13 (1.69 – 4.64)	1.71	4.64	0.12	Only compounds that were not measured with ammonia-based additives and not on pH = 2.91.	[13]

Table S2. Unified ionization efficiencies using the unified dataset. The table includes information about number of unique chemicals by InChI (#chemicals), number of measured datapoints under different conditions (#datapoints), number of chemicals common with dataset1 (#common with dataset1), number of chemicals common with unified by InChI and their logIE range (#common with unified), lowest and highest unified ionization efficiency value obtained after unification (Lowest logIE, Highest logIE), the variance between the common datapoints (σ^2), comments and references.

Dataset	#chemicals	#datapoints	#common with dataset1	#common with unified	Lowest logIE	Highest logIE	Variance (σ^2)	Comment on compounds	Ref.
Dataset10	50	196	6	21 (1.73 – 4.32)	1.73	4.31	0.05	Only compounds measured with “formic acid” as additive were considered, compounds “bicalutamide” and “17beta estradiol” were removed.	[13]
Dataset11	45	45	2	21 (0.68 – 3.21)	0.74	3.09	0.21		unpublished
Dataset12	14	68	7	8 (1.16 – 4.07)	1.27	4.01	0.55		[11]
Dataset13	24	26	1	20 (1.81 – 4.01)	2.10	3.91	0.28	Compound “chlorpyriphos” was removed.	unpublished

Table S3. All trained models' performance summary.

			xgbTree	*xgbLinear*	*xgbDART*
PaDEL	Training	RMSE =	0.557	0.328	0.577
		Q^2 =	0.461	0.436	0.487
	Test	RMSE =	0.805	0.866	0.725
		Mean =	11.660	14.765	11.247
		Geom. Mean =	4.412	4.891	3.785
		Median =	3.615	3.937	3.201
MS2Quant	Training	RMSE =	0.551	0.344	0.504
		Q^2 =	0.503	0.514	0.500
	Test	RMSE =	0.803	0.817	0.845
		Mean =	15.374	16.261	21.938
		Geom. Mean =	4.283	4.367	4.581
		Median =	3.246	3.269	3.540
Mordred	Training	RMSE =	0.764	0.385	0.846
		Q^2 =	0.059	0.044	0.068
	Test	RMSE =	1.169	1.405	1.127
		Mean =	59.433	168.801	53.488
		Geom. Mean =	9.141	13.058	8.457
		Median =	7.474	7.688	6.338
ECFP2	Training	RMSE =	1.005	0.522	0.958
		Q^2 =	0.159	0.133	0.176
	Test	RMSE =	1.109	1.203	1.092
		Mean =	57.396	379.350	63.225
		Geom. Mean =	7.927	9.146	7.664
		Median =	6.128	6.715	6.135
MAP4	Training	RMSE =	0.575	0.336	0.539
		Q^2 =	0.307	0.305	0.314
	Test	RMSE =	1.067	1.016	0.990
		Mean =	40.263	32.399	25.788
		Geom. Mean =	6.805	6.594	6.120
		Median =	4.645	5.315	4.512

Chapter S2. Summary of all tested descriptors

In addition to structural fingerprints used in MS2Quant, four different sets of molecular descriptors were tested for modelling ionization efficiencies: Pharmaceutical Data Exploration Laboratory (PaDEL) descriptors (1444 descriptors),[18] Mordred (1613 descriptors),[19] extended-connectivity fingerprints (ECFP) (1024 descriptors),[20] and minHashed atom-pair fingerprints (MAP4) (1024 descriptors).[21] PaDEL descriptors is a large set of continuous open-source 2D and 3D descriptors and structural fingerprints developed with the aim to be easily integrated to quantitative structure-activity relationship (QSAR) models. The wide range of descriptors include atom and bond counts, but also more complex descriptors, for example hydrogen bond donor and acceptor count and eccentric connectivity index.[18] Mordred descriptors are based on the PaDEL descriptors and attempt to overcome shortcomings of the latter with improved calculation algorithms.[19] ECFP are using the Morgan algorithm describing each atom as a central starting point, and in case of a radius of two (EFCP2), all atoms that are up to two bond lengths from the central atom.[20] As these hashed fingerprints describe relatively small part of a molecule in detail, they have been shown to work well for small compounds.[22,23] MinHashed atom-pair fingerprints with diameter up to four bonds (MAP4) utilize simultaneously ECFP as well as atom-pair fingerprints, which are effectively mapping large molecules and, therefore, have potential to working well over a large range of compounds with different sizes.[21]

The models using Morgan-2, MAP4, and Mordred descriptors were over-trained in spite of careful cross-validation for hyperparameter selection, and the predictive power on the test set was low with RMSE of test set values between 0.99 to 1.41 log-units. Therefore, these descriptors were not investigated further for quantification in NTS. The best performing algorithm throughout descriptors was *xgbTree*. The runner-up *xgbDART* algorithm yielded similar performance, but the errors were slightly bigger for MS2Quant. The *xgbLinear* algorithm-based models were more prone to overfitting, indicated by over two-fold difference in RMSE values of training and test set.

Chapter S3. NORMAN interlaboratory comparison and compounds

An interlaboratory comparison on quantification methods initiated by NORMAN Network was carried out by Malm et al. in 2021/2022[24]. In the study, water samples spiked with calibration and suspect compounds were analyzed by various laboratories using different LC-HRMS instruments and methods. In short, the calibration and suspect compounds used in this study had been selected from NORMAN Substance Database[25] representing both high and low ionization efficiency compounds and covering a wide chromatographic range. The set consisted of 41 calibration compounds and 44 suspect compounds, forming protonated molecules or molecular ions. All samples were spiked with three isotope labelled chemicals to check for systematic effects in between samples. No systematic errors were observed; therefore, isotope correction was not performed. For the interlaboratory study, samples containing calibration compounds were prepared in six concentrations in HPLC water (Honeywell Riedel-de Haën) and the samples containing suspect compounds were prepared in two concentrations in three different matrices (HPLC water, tap water and surface water collected from the lake Drevviken in Sweden).

The calibration samples and lake water samples spiked with suspect compounds in high and low concentration prepared in our lab were also analyzed and used in this study. Protonated species were observed for 36 and 39 compounds for which concentrations ranged from 8.5×10^{-10} to 6.3×10^{-6} M and 6.6×10^{-9} to 2.9×10^{-6} M for calibrants and suspects, respectively. The samples were measured with Dionex UltiMate™ 3000 ultra-high performance (UHP)LC system, coupled with a Q Exactive Orbitrap HRMS (Thermo Fischer Scientific™, Bremen, Germany). The following LC conditions were used: eluent A water with 0.1% formic acid, eluent B acetonitrile, flow rate 0.35 mL/min. The gradient started with 5% eluent B increasing to 100% eluent B in 20 minutes. It was held at 100% eluent B for 5 minutes before it decreased back to 5% eluent B in 0.1 minutes. The column (Kinetex 2.6 m EVO C18, 150×3.0 mm from Phenomenex, Torrence, CA, USA) was equilibrated for 5 minutes between each injection.

The ionization source settings used were spray voltage: 3.5 kV, max spray current: 100 A, capillary temperature: 320°C, S-lens RF level: 50%, aux gas heater temperature: 320°C, sheath gas flow rate: 35, aux gas flow rate: 3, sweep gas flow rate: 0 (all gas flow rates in arbitrary units).

To acquire MS^1, the following parameters were used: number of microscans: 1, resolution: 120 000, AGC target: 3×10^6, maximum IT: 200 ms. Two scans ranges (60.0000 – 900.0000 *m/z* and 100.0000 – 1500.0000 *m/z*) were used to acquire the MS^1 data. The MS^2 spectra were acquired in data dependent mode with where number of microscans: 1, resolution: 30 000, AGC target: 1×10^5, maximum IT: 60 ms and with an inclusion list of all protonated or positively charged suspect compounds, see SI. Stepwise normalized collision energies of 20, 70 and 120 were used for fragmentation. All measurements were performed in positive ESI mode.

Table S4. Calibration compounds and isotope labelled standards with molecular formula, theoretical ion mass, and the corresponding observed ion.

Chemical	Molecular formula	Theoretical ion mass	Observed ion
Amitrole	C2H4N4	85.0509	M+H+
Aspartame	C14H18N2O5	295.1288	M+H+
Atrazine	C8H14ClN5	216.1010	M+H+
Avermectin B1a H	C48H72O14	873.4995	M+H+
Benzotriazole	C6H5N3	120.0556	M+H+
Butocarboxim H	C7H14N2O2S	191.0849	M+H+
Caffeine	C8H10N4O2	195.0877	M+H+
Carbamazepine	C15H12N2O	237.1022	M+H+
Cefoperazone	C25H27N9O8S2	646.1497	M+H+
Chlormequat	C5H13ClN+	122.0731	M+
Chlorothiazide	C7H6ClN3O4S2	295.9561	M+H+
Clarithromycin H	C38H69NO13	748.4842	M+H+
Dichlorvos	C4H7Cl2O4P	220.9532	M+H+
Dimethyl phthalate	C10H10O4	163.0390	M+H-CH4O+
Diphenyl phthalate	C20H14O4	225.0546	M+H-C6H6O+
Efavirenz	C14H9ClF3NO2	316.0347	M+H+
Emamectin B1a	C49H75NO13	886.5311	M+H+
Guanylurea	C2H6N4O	103.0614	M+H+
Haloperidol	C21H23ClFNO2	376.1474	M+H+
Histamine	C5H9N3	112.0869	M+H+
Imazalil	C14H14Cl2N2O	297.0556	M+H+
Ivermectin B1a H	C48H74O14	875.5151	M+H+
L-alanine	C3H7NO2	90.05495	M+H+
L-phenylalanine	C9H11NO2	166.0863	M+H+
Methamidophos	C2H8NO2PS	142.0086	M+H+
Metolachlor	C15H22ClNO2	284.1412	M+H+
Nigericin H	C40H68O11	725.4834	M+H+
Octocrylene	C24H27NO2	362.2115	M+H+
Progesterone	C21H30O2	315.2319	M+H+
Rifaximin	C43H51N3O11	786.3596	M+H+
Saccharin	C7H5NO3S	184.0063	M+H+
Simazine	C7H12ClN5	202.0854	M+H+
Spinosad A	C41H65NO10	732.4681	M+H+
Sucralose H	C12H19Cl3O8	397.0218	M+H+
TCMTB	C9H6N2S3	238.9765869	M+H+
Tetraethylammonium	C8H20N+	130.1590261	M+
Tetrahexylammonium	C24H52N+	354.4094271	M+
Trichlorfon	C4H8Cl3O4P	256.9298548	M+H+
Tylosin	C46H77NO17	916.5264265	M+H+
Uracil	C4H4N2O2	113.0345538	M+H+
Vancomycin H	C66H75Cl2N9O24	1448.437476	M+H+
Atrazine-d_5	C8H14ClN5	220.1252	M+H+
Caffeine-$^{13}C_3$	C8H10N4O2	197.0904	M+H+
Haloperidol-d_4	C21H23ClFNO2	379.1652	M+H+

Table S5. Suspect compounds with molecular formula, theoretical ion mass, the corresponding observed ion, and the ranking of the correct structure from identification workflow.

Chemical	Molecular formula	Theoretical ion mass	Observed ion	Correct structure in identification found as following rank
10,11-dihydro-10-hydroxycarbamazepine	C15H14N2O2	255.1128	M+H+	1
2-(methylthio)benzothiazole	C8H7NS2	182.0093	M+H+	1
2-aminobenzothiazole	C7H6N2S	151.0324	M+H+	1
2-hydroxybenzothiazole	C7H5NOS	152.0165	M+H+	1
2-methylbenzothiazole	C8H7NS	150.0372	M+H+	1
5-chlorobenzotriazole	C6H4ClN3	154.0167	M+H+	2
5-methyl-1H-benzotriazole	C7H7N3	134.0713	M+H+	1
Acephate	C4H10NO3PS	184.0192	M+H+	1
Adenosine	C10H13N5O4	268.1040	M+H+	1
Ampicillin	C16H19N3O4S	350.1169	M+H+	1
Atrazine-2-OH	C8H15N5O	198.1349	M+H+	1
Atrazine-desethyl	C6H10ClN5	188.0697	M+H+	1
Atrazine-desethyl-2-OH	C6H11N5O	170.1036	M+H+	1
Atrazine-desethyl-desisopropyl	C3H4ClN5	146.0228	M+H+	1
Atrazine-desethyl-desisopropyl-2-OH	C3H5N5O	128.0567	M+H+	1
Atrazine-desisopropyl	C5H8ClN5	174.0541	M+H+	1
Atrazine-desisopropyl-2-OH	C5H9N5O	156.0880	M+H+	1
Benzothiazole	C7H5NS	136.0215	M+H+	1
Benzotriazole-5-carboxylic acid	C7H5N3O2	164.0455	M+H+	1
Butylamine	C4H11N	74.0964	M+H+	1
Carbamazepine-10,11-epoxide	C15H12N2O2	253.0972	M+H+	2
Chlorpyrifos	C9H11Cl3NO3PS	349.9336	M+H+	1
Climbazole	C15H17ClN2O2	293.1051	M+H+	1
Clotrimazole	C22H17ClN2	345.1153	M+H+	1
Dazomet	C5H10N2S2	163.0358	M+H+	3
Irgarol	C11H19N5S	254.1434	M+H+	1
Ketoconazole	C26H28Cl2N4O4	531.1560	M+H+	1
Melamine	C3H6N6	127.0727	M+H+	1
Metazachlor	C14H16ClN3O	278.1055	M+H+	1
Metformin	C4H11N5	130.1087	M+H+	1
Methidathion	C6H11N2O4PS3	302.9691	M+H+ +insource fragment	1
Methomyl	C5H10N2O2S	163.0536	M+H+	1
Metolachlor-ESA	C15H23NO5S	330.1370	M+H+	1
Metolachlor-OA	C15H21NO4	280.1543	M+H+	1
Monuron	C9H11ClN2O	199.0633	M+H+	1
Omethoate	C5H12NO4PS	214.0297	M+H+	1
Phenazine	C12H8N2	181.0760	M+H+	1
Reserpine	C33H40N2O9	609.2807	M+H+	1
Sebuthylazine	C9H16ClN5	230.1167	M+H+	3
Simazine-2-OH	C7H13N5O	184.1193	M+H+	1
Simvastatin H	C25H38O5	419.2792	M+H+	1
Sudan I	C16H12N2O	249.1022	M+H+	223
Theophylline	C7H8N4O2	181.0720	M+H+	1
Thiabendazole	C10H7N3S	202.0433	M+H+	1

Table S6. Summary table of the performance of all tested models on validation set. MS2Quant quantification is performed using MS^2 as well as based on structure; two PaDEL based models that use structure for quantification.

		MS2Quant (MS^2)	MS2Quant (structure)	PaDEL based model developed here (954 chemicals)	PaDEL based model developed by Liigand et al.[16] (353 chemicals)
Results of "true NTS" (MS2Quant from MS^2, others with top suggested structure) (39 chemicals)	RMSE	5.85	7.29	7.42	7.26
	R^2	0.46	0.37	0.47	0.49
	Mean	7.40	9.51	9.51	8.99
	Geom. mean	4.45	5.44	5.63	5.40
	Q25	2.16	2.48	2.29	2.27
	Q50 (Median)	4.02	4.57	5.19	4.87
	Q75	8.27	10.54	12.74	13.08
	Q90	17.43	25.31	26.09	20.36
	Q100 (Max)	47.68	55.26	54.91	45.87
Correct SMILES is used for quantifying suspects (39 chemicals)	RMSE		6.77	7.05	7.99
	R2		0.42	0.54	0.46
	Mean		8.18	8.61	9.89
	Geom. mean		5.29	5.44	6.00
	Q25		2.42	2.60	2.35
	Q50 (Median)		5.78	5.49	6.56
	Q75		9.71	9.47	15.20
	Q90		20.78	23.69	20.87
	Q100 (Max)		38.73	35.87	55.26
Only suspects that were correctly identified (34 chemicals)	RMSE	6.12	7.57	7.64	7.34
	R2	0.43	0.34	0.44	0.48
	Mean	7.80	9.91	9.81	8.83
	Geom. mean	4.67	5.69	5.83	5.52
	Q25	2.28	2.53	2.37	2.30
	Q50 (Median)	4.09	5.27	5.20	6.61
	Q75	8.36	10.67	13.35	13.09
	Q90	17.95	25.78	26.09	19.48
	Q100 (Max)	47.68	55.26	54.91	41.25
Only suspects that were incorrectly identified (5 chemicals)	RMSE	4.15	5.51	6.02	6.73
	R2	0.68	0.61	0.65	0.55
	Mean	4.66	6.78	7.46	10.09
	Geom. mean	3.20	4.00	4.47	4.63
	Q25	1.72	1.99	1.71	2.29
	Q50 (Median)	2.45	2.96	5.12	3.45
	Q75	4.70	6.68	6.41	4.91
	Q90	11.02	17.64	19.24	32.19
	Q100 (Max)	15.71	25.14	27.42	45.87
Only incorrectly identified suspects, but the correct SMILES was used for quantification (5 chemicals)	RMSE		4.39	6.29	6.77
	R2		0.59	0.72	0.54
	Mean		4.93	7.06	8.52
	Geom. mean		3.38	4.90	5.41
	Q25		1.84	2.00	2.67
	Q50 (Median)		2.16	6.94	4.69
	Q75		6.14	9.46	7.64
	Q90		11.04	12.97	21.90
	Q100 (Max)		15.73	18.48	31.21

Table S7. Calibration and summary plots of the performance of all tested models on validation set. MS2Quant quantification is performed using MS^2 as well as based on structure; two PaDEL based models that use structure for quantification.

	MS2Quant (MS^2)	MS2Quant (structure)	PaDEL based model developed here (954 chemicals)	PaDEL based model developed by Liigand et al.[16] (353 chemicals)
Calibration graph	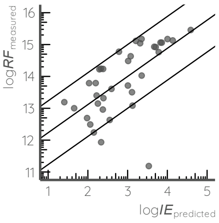<br>Residual standard error: 0.8523 on 34 degrees of freedom<br>Multiple R-squared: 0.3951,<br>Adjusted R-squared: 0.3773<br>F-statistic: 22.21 on 1 and 34 DF,<br>p-value: 4.038e-05		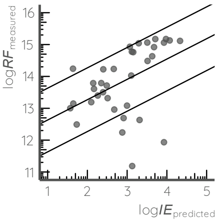<br>Residual standard error: 0.9521642 on 34 degrees of freedom<br>Multiple R-squared: 0.245036,<br>Adjusted R-squared: 0.2228311<br>F-statistic: 11.03526 on 1 and 34 DF, p-value: 0.00214551	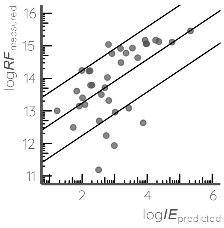<br>Residual standard error: 0.8975569 on 34 degrees of freedom<br>Multiple R-squared: 0.3291483,<br>Adjusted R-squared: 0.3094173<br>F-statistic: 16.68184 on 1 and 34 DF, p-value: 0.0002541113
Results of "true NTS" (MS2Quant from MS^2, others with top suggested structure) (39 chemicals)	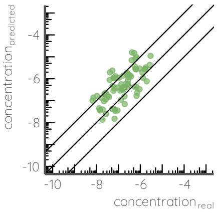	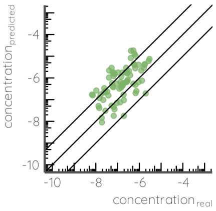	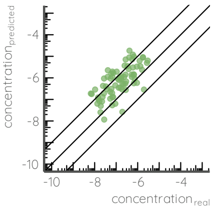	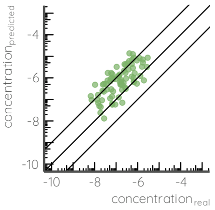
Correct SMILES is used for quantifying suspects (39 chemicals)		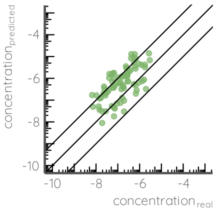	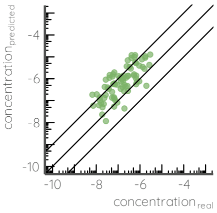	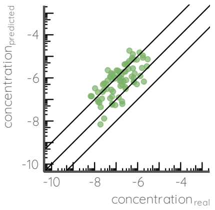
Only all suspects that were correctly identified (34 chemicals)	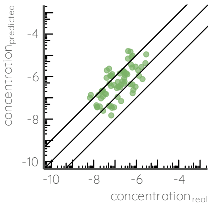	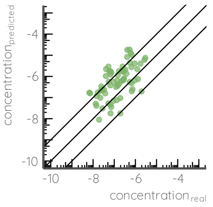	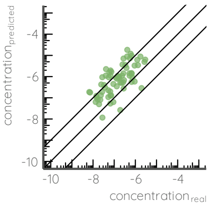	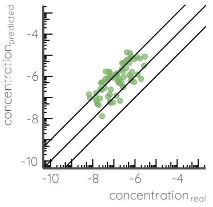
Only all suspects that were incorrectly identified (5 chemicals)	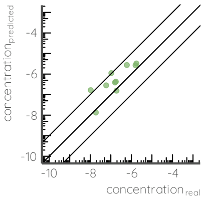	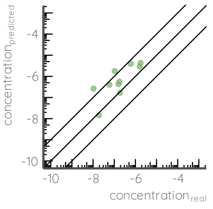	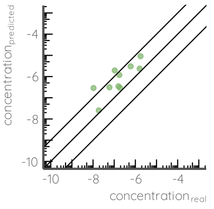	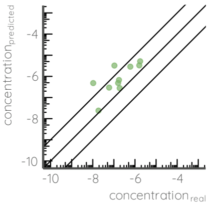
Only incorrectly identified compounds but the correct SMILES were used for quantification		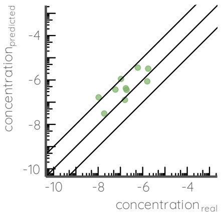	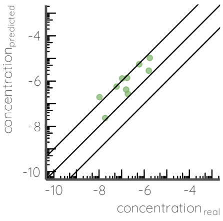	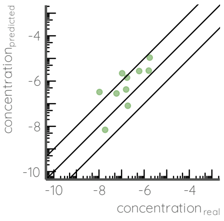

Table S8. Falsely identified compounds and their top suggested structure in identification workflow.

Correct structure	Top 1 match with SIRIUS+CSI:fingerID[26–30]
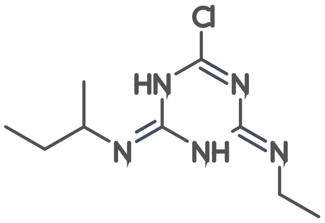 Sebuthylazine	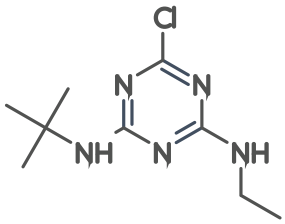 Terbuthylazine
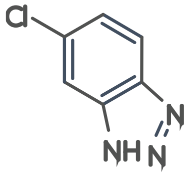 5-chlorobenzotriazole	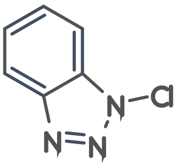 1-Chlorobenzotriazole
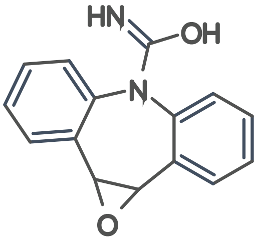 Carbamazepine-10,11-epoxide	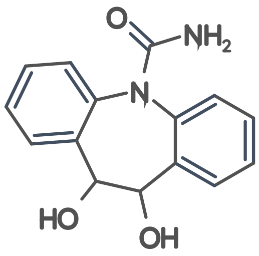 10,11-Dihydroxycarbamazepine
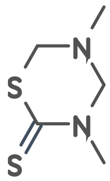 Dazomet	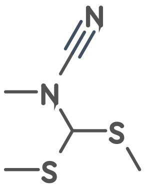 Bis(methylsulfanyl)methyl-methylcyanamide
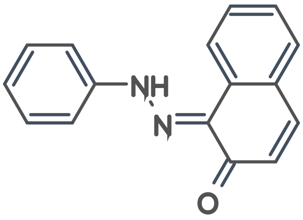 Sudan I	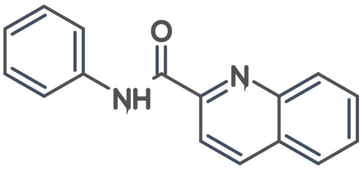 Quinaldanilide

Chapter S4. SIRIUS+CSI:FingerID for calculating structural fingerprints and identification

For data processing, MSDIAL was used to extract *.ms* files for all suspected chemicals from data-dependent analysis with inclusion lists. Detected suspect LC-HRMS peaks without MS^2 spectrum were discarded. Additional filtering was done based on previously determined retention times and exact mass of the detected ion, which resulted in unique *.ms* files for 41 suspect compounds.

SIRIUS+CSI:FingerID (version 4.9.15)[26–30] was used to calculate probabilities of structural fingerprints from *.ms* files containing MS^1 and MS^2 information. Based on the spectra, fragmentation trees were calculated where peaks were annotated with potential fragments and connected with assumed losses, then kernel similarities were calculated against all reference compounds. Previously trained support vector machines were used to predict the probability for each molecular property, and for each chemical a vector of probabilities for each molecular property was obtained. As probabilities of fingerprints are calculated using pre-trained models, no databases are required for calculations; however, it is possible to use all included databases to only consider logical molecular formulas and their fragmentation trees for predicting fingerprints. The latter was used in this work. The vector of probabilities for each compound was compared against all databases available in SIRIUS for identification. For all compounds in the database, the probabilities of each molecular fingerprint were calculated from the structure, where presence or absence can be determined with full certainty resulting binary probability values (either 0 or 1).[28] The top highest ranked structure for each LC-HRMS peak was assigned as the tentative structure and will further be referred to as top structural candidate. The structural fingerprints that remained after data preprocessing and were used for modelling can also be calculated with the latest SIRIUS version 5.6.2.

SIRIUS+CSI:FingerID calculations were conducted using following conditions: infinite number of all default elements (H, C, N, O, P, B, Si, S, Cl, Se, Br, Fl and I) were allowed in formula prediction, “orbitrap” was used as instrument with mass deviation of 5 ppm and matches for formulas and structures were searched in all available databases.

Table S9. Top 10 most influential variables learned by PaDEL based model developed here.

Feature	Relative importance	Descriptor class	Description
VR1_D	100.00	Topological Distance Matrix descriptor	Randic-like eigenvector-based index from topological distance matrix
ATSC0i	90.96	Auto correlation descriptor	Centered Broto-Moreau autocorrelation - lag 0 / weighted by first ionization potential
AATSC0e	78.70	Auto correlation descriptor	Average centered Broto-Moreau autocorrelation - lag 0 / weighted by Sanderson electronegativities
pH_aq	30.90	Eluent descriptor	Aqueous pH
GATS2s	29.72	Auto correlation descriptor	Geary autocorrelation - lag 2 / weighted by I-state
C2SP3	29.09	Carbon types descriptor	Singly bound carbon bound to two other carbons
ATSC7i	19.88	Auto correlation descriptor	Centered Broto-Moreau autocorrelation - lag 7 / weighted by first ionization potential
polarity_index	18.97	Eluent descriptor	Polarity index
nN	18.83	Atom count descriptor	Number of nitrogen atoms
MLFER_BH	17.14	Molecular linear free energy relation	Overall or summation solute hydrogen bond basicity

Figure S2. PaDEL-based model analysis. A) Top 10 most influential variables in the PaDEL based model and their normalized importance (%); B) SHAP values representing influence of each top 10 feature and their marginal contribution to the prediction and C) the test set chemicals assigned to different classes by ClassyFire, where each datapoint represents the geometric mean prediction error of log*IE* of a unique chemical. The classes are in descending order based on median geometric mean prediction error of all compounds in the group and only classes with three or more unique representatives were plotted.

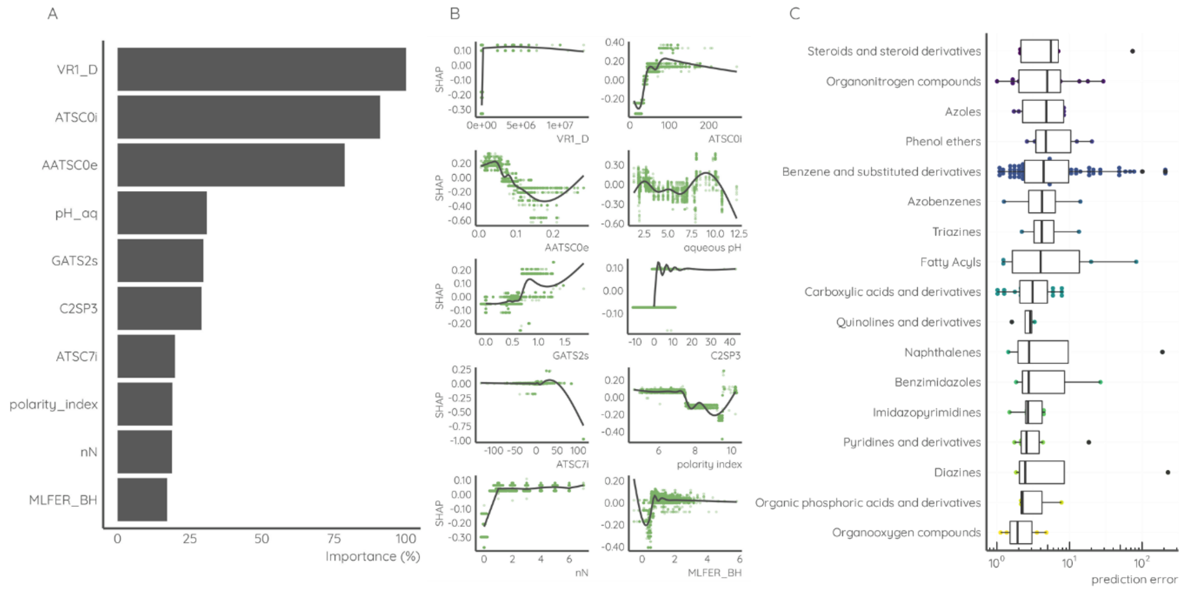

Figure S3. First tree of xgbTree models for A) MS2Quant and B) PaDEL based model developed here.

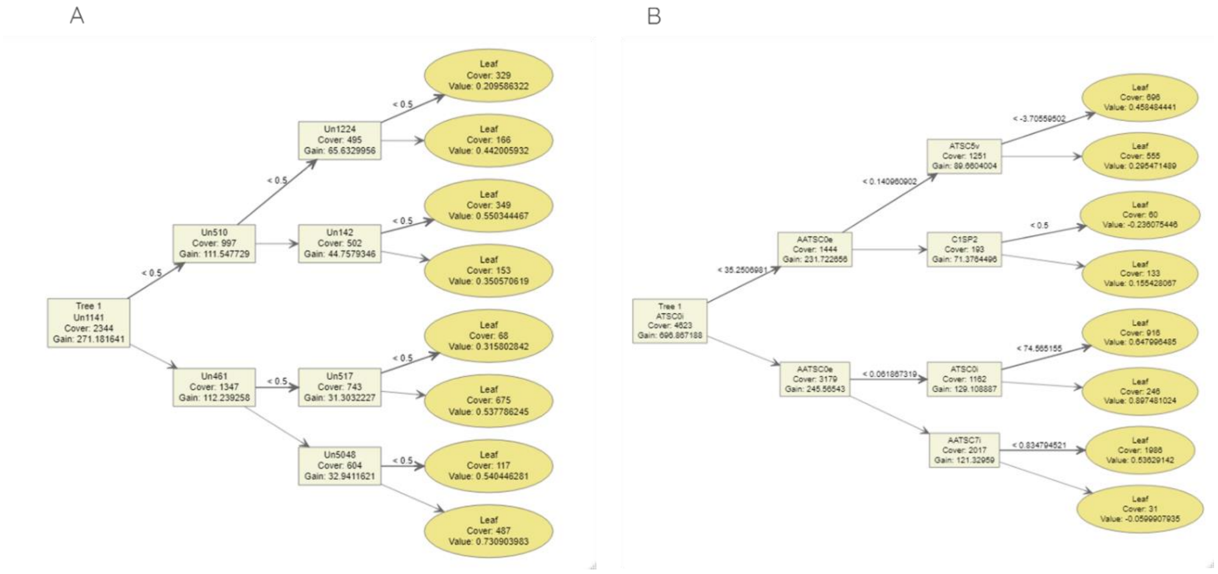

References

1. Oss, M., Kruve, A., Herodes, K. & Leito, I. Electrospray Ionization Efficiency Scale of Organic Compounds. *Anal. Chem.* 82, 2865–2872 (2010).
2. Kruve, A. & Kaupmees, K. Adduct Formation in ESI/MS by Mobile Phase Additives. *J. Am. Soc. Mass Spectrom.* 28, 887–894 (2017).
3. Liigand, J., Laaniste, A. & Kruve, A. pH Effects on Electrospray Ionization Efficiency. *J. Am. Soc. Mass Spectrom.* 28, 461–469 (2017).
4. Liigand, J., Kruve, A., Leito, I., Girod, M. & Antoine, R. Effect of Mobile Phase on Electrospray Ionization Efficiency. *J. Am. Soc. Mass Spectrom.* 25, 1853–1861 (2014).
5. Ojakivi, M., Liigand, J. & Kruve, A. Modifying the Acidity of Charged Droplets. *ChemistrySelect* 3, 335–338 (2018).
6. Kruve, A., Kiefer, K. & Hollender, J. Benchmarking of the quantification approaches for the non-targeted screening of micropollutants and their transformation products in groundwater. *Anal. Bioanal. Chem.* 413, 1549–1559 (2021).
7. Costalunga, R., Tshepelevitsh, S., Sepman, H., Kull, M. & Kruve, A. Sodium adduct formation with graph-based machine learning can aid structural elucidation in non-targeted LC/ESI/HRMS. *Anal. Chim. Acta* 1204, 339402 (2022).
8. Liigand, J., de Vries, R. & Cuyckens, F. Optimization of flow splitting and make-up flow conditions in liquid chromatography/electrospray ionization mass spectrometry. *Rapid Commun. Mass Spectrom.* 33, 314–322 (2019).
9. Liigand, P. *et al.* Think Negative: Finding the Best Electrospray Ionization/MS Mode for Your Analyte. *Anal. Chem.* 89, 5665–5668 (2017).
10. Gornischeff, A., Kruve, A. & Rebane, R. Characterization of wines with liquid chromatography electrospray ionization mass spectrometry: Quantification of amino acids via ionization efficiency values. *J. Chromatogr. A* 1620, 461012 (2020).

11. Sepman, H., Tshepelevitsh, S., Hupatz, H. & Kruve, A. Protomer Formation Can Aid the Structural Identification of Caffeine Metabolites. *Anal. Chem.* 94, 10601–10609 (2022).
12. Wang, T., Liigand, J., Frandsen, H. L., Smedsgaard, J. & Kruve, A. Standard substances free quantification makes LC/ESI/MS non-targeted screening of pesticides in cereals comparable between labs. *Food Chem.* 318, 126460 (2020).
13. Souihi, A., Mohai, M. P., Palm, E., Malm, L. & Kruve, A. MultiConditionRT: Predicting liquid chromatography retention time for emerging contaminants for a wide range of eluent compositions and stationary phases. *J. Chromatogr. A* 1666, 462867 (2022).
14. Malm, L. *et al.* Guide to Semi-Quantitative Non-Targeted Screening Using LC/ESI/HRMS. *Molecules* 26, 3524 (2021).
15. Liigand, J. *et al.* Transferability of the Electrospray Ionization Efficiency Scale between Different Instruments. *J. Am. Soc. Mass Spectrom.* 26, 1923–1930 (2015).
16. Liigand, J. *et al.* Quantification for non-targeted LC/MS screening without standard substances. *Sci. Rep.* 10, 5808 (2020).
17. Ulrich, E. M. *et al.* EPA's non-targeted analysis collaborative trial (ENTACT): genesis, design, and initial findings. *Anal. Bioanal. Chem.* 411, 853–866 (2019).
18. Yap, C. W. PaDEL-descriptor: An open source software to calculate molecular descriptors and fingerprints. *J. Comput. Chem.* 32, 1466–1474 (2011).
19. Moriwaki, H., Tian, Y.-S., Kawashita, N. & Takagi, T. Mordred: a molecular descriptor calculator. *J. Cheminformatics* 10, 4 (2018).
20. Rogers, D. & Hahn, M. Extended-Connectivity Fingerprints. *J. Chem. Inf. Model.* 50, 742–754 (2010).
21. Capecchi, A., Probst, D. & Reymond, J.-L. One molecular fingerprint to rule them all: drugs, biomolecules, and the metabolome. *J. Cheminformatics* 12, 43 (2020).

22. Bach, E., Schymanski, E. L. & Rousu, J. Joint structural annotation of small molecules using liquid chromatography retention order and tandem mass spectrometry data. *Nat. Mach. Intell.* 4, 1224–1237 (2022).
23. Achebouche, R., Tromelin, A., Audouze, K. & Taboureau, O. Application of artificial intelligence to decode the relationships between smell, olfactory receptors and small molecules. *Sci. Rep.* 12, 18817 (2022).
24. Kruve, A., Aalizadeh, R., Malm, L., Alygizakis, N. & Thomaidis, N. S. Interlaboratory Comparison on Strategies for Semi-Quantitative Non-Targeted LC-ESI-HRMS. (2020).
25. NORMAN Network *et al.* S0 | SUSDAT | Merged NORMAN Suspect List: SusDat. (2022) doi:10.5281/ZENODO.2664077.
26. Hoffmann, M. A. *et al.* High-confidence structural annotation of metabolites absent from spectral libraries. *Nat. Biotechnol.* 40, 411–421 (2022).
27. Heinonen, M., Shen, H., Zamboni, N. & Rousu, J. Metabolite identification and molecular fingerprint prediction through machine learning. *Bioinformatics* 28, 2333–2341 (2012).
28. Dührkop, K., Shen, H., Meusel, M., Rousu, J. & Böcker, S. Searching molecular structure databases with tandem mass spectra using CSI:FingerID. *Proc. Natl. Acad. Sci.* 112, 12580–12585 (2015).
29. Böcker, S. & Dührkop, K. Fragmentation trees reloaded. *J. Cheminformatics* 8, 5 (2016).
30. Dührkop, K. *et al.* SIRIUS 4: a rapid tool for turning tandem mass spectra into metabolite structure information. *Nat. Methods* 16, 299–302 (2019).
